# Economic evaluation of postdischarge malaria chemoprevention in preschool children treated for severe anaemia in Malawi, Kenya, and Uganda: A cost-effectiveness analysis

**DOI:** 10.1016/j.eclinm.2022.101669

**Published:** 2022-10-01

**Authors:** Melf-Jakob Kühl, Thandile Gondwe, Aggrey Dhabangi, Titus K. Kwambai, Amani T. Mori, Robert Opoka, C. Chandy John, Richard Idro, Feiko O. ter Kuile, Kamija S. Phiri, Bjarne Robberstad

**Affiliations:** a Centre for International Health (CIH), Department of Global Public Health and Primary Care, University of Bergen, Årstadveien 17, 5009 Bergen, Norway; b Kamuzu University of Health Sciences, 782 Mahatma Gandhi, Blantyre, Malawi; c Training and Research Unit of Excellence, 1 Kufa Road, Blantyre, Malawi; d Makerere University College of Health Sciences, Upper Mulago Hill Road, Kampala, Uganda; e Centre for Global Health Research (CGHR), Kenya Medical Research Institute (KEMRI), Busia Rd, Kisumu, Kenya; f Department of Clinical Sciences, Liverpool School of Tropical Medicine (LSTM), Pembroke Place, Liverpool L3 5QA, United Kingdom; g Chr. Michelsen Institute, Jekteviksbakken 31, 5006 Bergen, Norway; h Ryan White Center for Pediatric Infectious Diseases and Global Health, School of Medicine, Indiana University, 1044 W Walnut St, R4 402D Indianapolis, United States of America; i Health Economics Leadership and Translational Ethics Research Group (HELTER), Department of Global Public Health and Primary Care, University of Bergen, Årstadveien 17, 5009 Bergen, Norway

**Keywords:** Economic evaluation, Cost-effectiveness analysis, Intermittent preventive therapy, IPTpd, Postdischarge, Post-discharge, malaria chemoprevention, PDMC, PMC, Malaria prophylaxis, malaria prevention, Dihydroartemisinin-piperaquine, DP, Adherence, Sub-Saharan Africa, Malawi, Kenya, Uganda, Preschool children, Children under five years of age, Severe anaemia

## Abstract

**Background:**

Children hospitalised with severe anaemia in malaria-endemic areas are at a high risk of dying or being readmitted within six months of discharge. A trial in Kenya and Uganda showed that three months of postdischarge malaria chemoprevention (PDMC) with monthly dihydroartemisinin-piperaquine (DP) substantially reduced this risk. The World Health Organization recently included PDMC in its malaria chemoprevention guidelines. We conducted a cost-effectiveness analysis of community-based PDMC delivery (supplying all three PDMC-DP courses to caregivers at discharge to administer at home), facility-based PDMC delivery (monthly dispensing of PDMC-DP at the hospital), and the standard of care (no PDMC).

**Methods:**

We combined data from two recently completed trials; one placebo-controlled trial in Kenya and Uganda collecting efficacy data (May 6, 2016 until November 15, 2018; n=1049), and one delivery mechanism trial from Malawi collecting adherence data (March 24, 2016 until October 3, 2018; n=375). Cost data were collected alongside both trials. Three Markov decision models, one each for Malawi, Kenya, and Uganda, were used to compute incremental cost-effectiveness ratios expressed as costs per quality-adjusted life-year (QALY) gained. Deterministic and probabilistic sensitivity analyses were performed to account for uncertainty.

**Findings:**

Both PDMC strategies were cost-saving in each country, meaning less costly and more effective in increasing health-adjusted life expectancy than the standard of care. The estimated incremental cost savings for community-based PDMC compared to the standard of care were US$ 22·10 (Malawi), 38·52 (Kenya), and 26·23 (Uganda) per child treated. The incremental effectiveness gain using either PDMC strategy varied between 0·3 and 0·4 QALYs. Community-based PDMC was less costly and more effective than facility-based PDMC. These results remained robust in sensitivity analyses.

**Interpretation:**

PDMC under implementation conditions is cost-saving. Caregivers receiving PDMC at discharge is a cost-effective delivery strategy for implementation in malaria-endemic southeastern African settings.

**Funding:**

Research Council of Norway.

**Research in context**

**Evidence before this study**

In malaria-endemic areas of sub-Saharan Africa preschool children treated for severe anaemia are 72% more likely to die within six months of discharge than during the in-hospital period. Three months post-discharge malaria chemoprevention (PDMC) reduces post-discharge mortality and hospital readmissions by 70%. In 20 high-burden African countries, one hospital readmission could be prevented for every two to five children receiving PDMC, amounting to an estimated 36 000 annual hospital readmissions averted under full PDMC coverage. Using the search terms “cost-effective*”, “cost-benefit”, or “economic evaluation” paired with “malaria”, “anaemia”, or “anemia”, with “post-discharge”, “post-discharge”, or “post-discharge” with “prophyla*” or “prevent*”, and with “child*”, we searched without language restriction for publications published between Jan 1, 2000, and Aug 25, 2022, in the databases of PubMed (seven results) and Web of Science (five results). We conducted the searches on Aug 26, 2022, and found no previous economic evaluations of post-discharge use of malaria chemoprevention in children.

**Added value of this study**

This study offers a methodological approach to combining cost information with adherence and efficacy data in country-specific Markov models. We show that implementing PDMC would be cost-saving and likely cost-effective in Kenya, Uganda, and Malawi. We identify a cost-effective delivery strategy: providing all PDMC courses to the caregiver at discharge to administer monthly at home.

**Implications of all the available evidence**

Countries in sub-Saharan Africa with moderate to high malaria transmission should consider making PDMC accessible to all children with severe anaemia surviving the acute in-hospital phase.

Alt-text: Unlabelled box

## Introduction

Despite large-scale control efforts, malaria burden reductions have stagnated in parts of sub-Saharan Africa.^1^ Severe anaemia remains a leading cause of mortality and morbidity in children under five years of age, and malaria is one of the main causes. In highly malaria-endemic areas, severe anaemia may be found in approximately one-third of hospitalised children and contribute to 50% of deaths attributed to malaria.^2^, ^3^, ^4^, ^5^, ^6^

Young children discharged from hospital after treatment for severe anaemia are at high risk of dying or being readmitted for at least six months postdischarge,^7^, ^8^, ^9^ this risk is 2·7 times higher than children admitted for other reasons and 1·7 times higher than during hospitalisation.^10^ In June 2022, postdischarge malaria chemoprevention (‘PDMC’, previously called ‘PMC’ and ‘IPTpd’) was included in the updated malaria chemoprevention guidelines from the World Health Organisation (WHO) for settings with moderate to high malaria transmission.^12^ This was based in part on the results of a multi-country trial in Kenya and Uganda that showed that in preschool children with severe anaemia, three months of monthly PDMC with the long-acting antimalarial dihydroartemisinin-piperaquine (DP) reduced the risk of malaria-associated re-admission or death by 70% during the three months intervention period.^8^ This suggests that malaria is a major cause of morbidity and mortality after discharge in these areas. An implementation trial in Malawi compared the effects of community-based versus facility-based delivery strategies for PDMC on adherence to all three courses of PDMC.^11^ The highest adherence was achieved with community-based delivery, where caregivers were provided at discharge with all courses to administer PDMC monthly at home. Both trials were performed simultaneously between 2016 and 2018.

Based on the WHO guidelines, countries in sub-Saharan Africa with moderate to high malaria transmission should consider making PDMC accessible to all children with severe anaemia surviving the acute in-hospital phase. Here we combined data from these two trials to establish the cost-effectiveness of PDMC under implementation conditions and inform national guideline development in malaria-endemic areas in sub-Saharan Africa.

## Methods

### Study design

Three novel decision-analytical discrete-time models (Markov), one each for Malawi, Kenya, and Uganda, were developed to assess the cost-effectiveness per country of the two PDMC delivery strategies against the standard of care using TreeAge Pro 2022. Results were reported according to the Consolidated Health Economic Evaluation Reporting Standards-statement.^13^ We combined data from the efficacy trial in Kenya and Uganda, data from the implementation trial in Malawi, data from interviews and process observations in Malawi, and data from the literature. Each country model used the same three health states: *healthy, severely sick*, and *dead,* with severe sickness defined as any hospital admission within six months of discharge. The modelled cohorts entered the model upon the first PDMC course, which was given approximately 14 days after discharge from the hospital. We assumed the cohort to start in the *healthy* state and then move within the model in six cycles of one month each. At the end of each cycle, children in the cohort could change between the *healthy* and *severely sick* states. The absorbing *dead* state could be reached from either the *healthy* or the *severely sick* state. Additionally, non-severe health events, mostly clinic visits for uncomplicated clinical malaria, were modelled as occurring during a cycle within the *healthy* state (Figure S1). We conducted deterministic and probabilistic sensitivity analyses for each country and reported the results as incremental cost-effectiveness ratios (ICERs) expressed as costs per quality-adjusted life-year (QALY) gained. We used 3% global discounting for all costs and utilities.

### Efficacy and adherence data

The efficacy estimates were obtained from the trial in Kenya and Uganda.^8^ This two-arm placebo-controlled trial used three courses of monthly PDMC regimen with DP administered at the ends of the 2nd, 6th, and 10th week postdischarge. Each course comprised three doses of DP given once daily. Adherence to the first dose of each monthly course was assessed during home visits as directly observed therapy. In addition, daily telephone contact with caregivers and random home visits were used to verify the adherence to each course's second and third dose. Mortality and readmission rates were assessed for six months postdischarge.

The adherence data were obtained from the trial in Malawi that assessed adherence to the same PDMC regimen and compared community-based with facility-based delivery strategies.^11^ Community-based PDMC consisted of providing all three PDMC courses to the caregivers at the time of hospital discharge combined with instructions how to administer the tablets at home. Facility-based PDMC consisted of instructions to the caregivers to collect each monthly DP course from the hospital's outpatient department. After each course, adherence was determined by inspection of blister packs collected during unannounced home visits. Community-based PDMC resulted in higher adherence than facility-based (71% vs 52% adherence to the full three courses, Table S1). We categorised adherence into *high* (all nine tablets taken, three per course), *medium* (six to eight tablets), *low* (three to five tablets), and *very low or no* adherence (zero to two tablets). We used these adherence rates to project the efficacy of PDMC under implementation conditions (Figure S2).

All study hospitals in Malawi and Uganda (public hospitals) and Kenya (public and private hospitals) were in high malaria transmission areas. Both trials included children aged younger than five years admitted for all-cause severe anaemia, excluding severe anaemia due to genetic factors, trauma, or malignancies. Hospitalised children received the standard of care for severe anaemia, including blood transfusions, parenteral antimalarials (in case of severe malarial anaemia), and antibiotics when indicated. At discharge, all children received the standard of care consisting of 3-day antimalarial treatment with oral artemether-lumefantrine, which provides an average of about 13 days of post-treatment prophylaxis against malaria, regardless of the presence of malaria parasites at the time of treatment.^14^

### Effects and rewards

Lacking quality of life weights, we used inverted annual disability weights from the 2019 Global Burden of Disease study to approximate QALYs for severe sickness and non-severe events.^15^^,^^16^ Within the first six months, completing a month in the *healthy* state was rewarded with the monthly equivalent of one full QALY. During this period, any hospital readmission (*severely sick*) translated to a one-month-long QALY reduction by the weighted average disability weight for the causes of readmission recorded in the efficacy trial (0·158 QALY/12). Based on the same data, any disutility from a non-severe health event within the healthy state was equated to two weeks of the average annual disability weight of these events (0·046 QALY/26). For children who died, no further QALYs were accounted. Based on our assumption of complete recovery by six months, surviving children were awarded their 2018 national average health-adjusted life expectancy subtracted by their average age at study completion (Malawi: 54·7 years; Kenya: 56·0 years; Uganda: 56·0 years).^17^ The rewards were not half-cycle-corrected because of the relatively short cycle length.

The monthly transitions between the three health states were controlled by transition probabilities extracted from the efficacy trial's health outcomes (Table S2).^8^ We assumed that the trial's outcomes for the PDMC-arm and for the placebo-arm corresponded to the efficacy of 100% and 0% adherence to PDMC, respectively. We further assumed a linear dose-response and matched the mean number of administered tablets per adherence category with the corresponding efficacy estimate. For example, *high adherence* (nine out of nine tablets taken) corresponded to 100% of the established efficacy, whereas for *medium adherence* (mean of 6·04 of nine tables given), we adjusted the efficacy by 67%. In this category, the modelled death or readmission probabilities were adjusted to combine 67% transition probabilities corresponding to the trial's PDMC arm with 33% of probabilities corresponding to the placebo arm. We repeated this process by linear interpolation for the other two adherence categories (Figure S2). We disregarded information about the order of courses in case of non-adherence, for example, whether the 1^st^, 2^nd^, or 3^rd^ course of PDMC was skipped in a child who received six out of nine tablets because no evidence existed how this impacted PDMC efficacy.

### Intervention costs

We combined the healthcare provider perspective with the patients’ household perspective to estimate the societal cost of PDMC implementation. We included both intervention-related costs and the costs of adverse health events during the discharge period. We employed a pragmatic ingredients approach, based on a mixed-methods inquiry, to determine directly and indirectly incurred costs related to PDMC and health outcomes postdischarge.^18^

We collected provider intervention cost data at Zomba Central Hospital in Malawi in 2018. For Kenya and Uganda, personnel salaries were based on local rates. We adopted providers’ cost of DP from the national procurement systems (Malawi) and the literature (Kenya, Uganda), with a 30% surcharge for handling and wastage as it is standard practise in Malawi (Tables 1 and S5). Pharmacies’ additional costs to disseminate and orient patients on PDMC in Malawi, according to the two PDMC strategies, were determined by time and motion observations and the average salaries of the involved personnel (Table S4). The intervention costs to households, i.e. the cost of receiving and administering DP, were prospectively collected alongside both trials and in the analysis adjusted to delivery strategy and strategy-dependent adherence rates (Table S7).

Both delivery strategies of PDMC started two weeks postdischarge. The baseline cost for the standard of care was incurred before starting the first postdischarge course of PDMC and was therefore assumed to be zero for all three arms. The intervention cost to the providers was estimated to be between 2·48 and 4·41 United States Dollars (USD) for either PDMC delivery strategy in any country (Table 1). In contrast, the baseline intervention costs to households differed substantially between the delivery arms and countries. Community-based delivery, i.e., receiving all three PDMC courses upon discharge with instructions on administering them, was estimated to cost caregivers an average of 0·26 USD in Malawi, and 0·09 and 0·07 USD in Kenya and Uganda, respectively. Facility-based delivery resulted in substantially higher costs incurred by households (7·43 USD in Malawi, 10·09 USD in Kenya, 10·16 USD in Uganda) due to the required travel to the hospital (Table 1, S7). The households’ costs to administer a PDMC course were assumed to be the same in both arms. The households’ lost productivity due to administering PDMC was estimated as the value of time spent providing the care. We valued the time using the minimum national salary rates of 2018. Direct and indirect costs were allowed to vary by country (Tables 1 and S7–8).

**Table 1:** Component costs and estimated distributions for postdischarge malaria chemoprevention (PDMC) interventions and adverse health events from provider and household perspectives for each country.

	Base Case			Range (CI*; point estimates: +/- 50%)				Distribution	Source
				low		high			
PERSPECTIVE: Cost component	Standard of care	PDMC Community delivery	PDMC Facility delivery	PDMC Community delivery	PDMC Facility delivery	PDMC Community delivery	PDMC Facility delivery		
**Intervention costs**									
PROVIDER: Dihydroartemisinin–piperaquine (DP) price per treatment, USD*									
Kenya	0·00	2·97	2·97	1·48		4·45		Point estimate	MSH Price Guide (2015)^19^
Malawi	0·00	2·36	2·36	1·18		3·54		Point estimate	Fernandes (2020)^20^
Uganda	0·00	2·30	2·30	1·15		3·45		Point estimate	GF Price Reference Report (2015)^21^
PROVIDER: Pharmacist time cost, USD									
Kenya	0·00	0·72	1·44	0·36	0·72	1·08	2·16	Point estimate	PDMC Malawi cost study (unpublished, Tables S3-9)
Malawi	0·00	0·12	0·24	0·06	0·12	0·18	0·36	Point estimate	
Uganda	0·00	0·29	0·58	0·15	0·29	0·44	0·87	Point estimate	
HOUSEHOLD: Total household drug collection cost, USD									
Kenya	0·00	0·09	10·09	0·05	5·05	0·14	15·13	Gamma	PDMC Malawi cost study (unpublished, Tables S3-9)
Malawi	0·00	0·26	7·43	0·13	3·72	0·39	11·15	Gamma	
Uganda	0·00	0·07	10·16	0·04	5·08	0·11	15·24	Gamma	
HOUSEHOLD: total household drug administration cost, USD									
Kenya	0·00	1·94		0·97		2·91		Gamma	Kwambai (2020)^8^
Malawi	0·00	1·07		0·54		1·61		Gamma	PDMC Malawi cost study (unpublished, Tables S3-9)
Uganda	0·00	3·31		1·66		4·97		Gamma	Kwambai (2020)^8^
**Costs of adverse health events**									
PROVIDER: total health personnel cost per inpatient treatment, USD									
Kenya	15·74			7·87		23·61		Point estimate	PDMC Malawi cost study (unpublished, Tables S3-9)
Malawi	10·56			5·28		15·84		Point estimate	
Uganda	11·45			5·73		17·18		Point estimate	
PROVIDER: cost per blood transfusions per inpatient treatment incl.· laboratory costs, transportation and wastage, USD									
Kenya	73·10			36·55		109·65		Point estimate	PDMC Malawi cost study (unpublished) combined with adjusted costs from Medina-Lara (2007)^22^
Malawi	65·93			32·97		98·60		Point estimate	
Uganda	82·24			41·12		123·36		Point estimate	
PROVIDER: sum of medicines, equipment, and other material costs per inpatient treatment, including SOC at discharge, USD									
Kenya	18·67			9·34		28·01		Point estimate	PDMC Malawi cost study (unpublished, Tables S3-9)
Malawi	18·67			9·34		28·01		Point estimate	
Uganda	18·67			9·34		28·01		Point estimate	
PROVIDER: sum of hospital administration and support services per inpatient treatment, USD									
Kenya	12·37			6·19		18·56		Point estimate	PDMC Malawi cost study (unpublished, Tables S3–9)
Malawi	3·04			1·52		4·56		Point estimate	
Uganda	5·17			2·59		7·76		Point estimate	
PROVIDER: total cost per outpatient treatment, USD									
Kenya	3·39			1·77		5·01		Point estimate	PDMC Malawi cost study (unpublished, Tables S3–9)
Malawi	2·40			1·22		3·58		Point estimate	
Uganda	2·53			1·31		3·75		Point estimate	
HOUSEHOLD: total cost per inpatient stay incl.· transport and lost productivity, and patient transfusion cost (only applicable for Kenya), USD									
Kenya	55·26			40·09		70·43		Gamma	Kwambai (2020)^8^
Malawi	12·94			11·35		14·54		Gamma	PDMC Malawi cost study (unpublished, Tables S3–9)
Uganda	20·04			18·10		21·98		Gamma	Kwambai (2020)^8^
HOUSEHOLD: total household cost per outpatient visit incl.· transport and lost productivity, USD									
Kenya	11·92			9·34		14·49		Gamma	Kwambai (2020)^8^
Malawi	5·34			4·31		6·70		Gamma	PDMC Malawi cost study (unpublished, Tables S3–9)
Uganda	9·60			8·45		10·75		Gamma	Kwambai (2020)^8^

The detailed items summarised in the component costs can be found in the supplementary materials (*Tables S5, S10*).

*CI– Confidence interval (95%).

**USD– United States Dollar.

### Costs of adverse health events

We assumed the cost per hospital readmission after discharge to be generally the same in all arms and that they only differed by country. As a proxy for the provider and household costs for any “all-cause” readmission, we used the average costs incurred for treating severe anaemia at Zomba Central Hospital, Malawi. Patient and clinical pathways were recorded by following clinical practice and interviewing hospital staff. The costs of involved personnel were calculated based on hospitals’ average salaries for these positions and the reported time spent per patient (Table S7). Fifty random treatment records of children enrolled in the implementation trial in Malawi were reviewed for readmission duration, medication and procedures provided. The costs of medicines and equipment were itemised, valued, and costed based on Malawi's central health equipment procurement database.^23^ Extra costs for handling and wastage were also added (Table S6). These costs were adopted for the Kenyan and Ugandan models. We excluded all costs related to a child's death, such as funeral costs.

**Figure 1:**
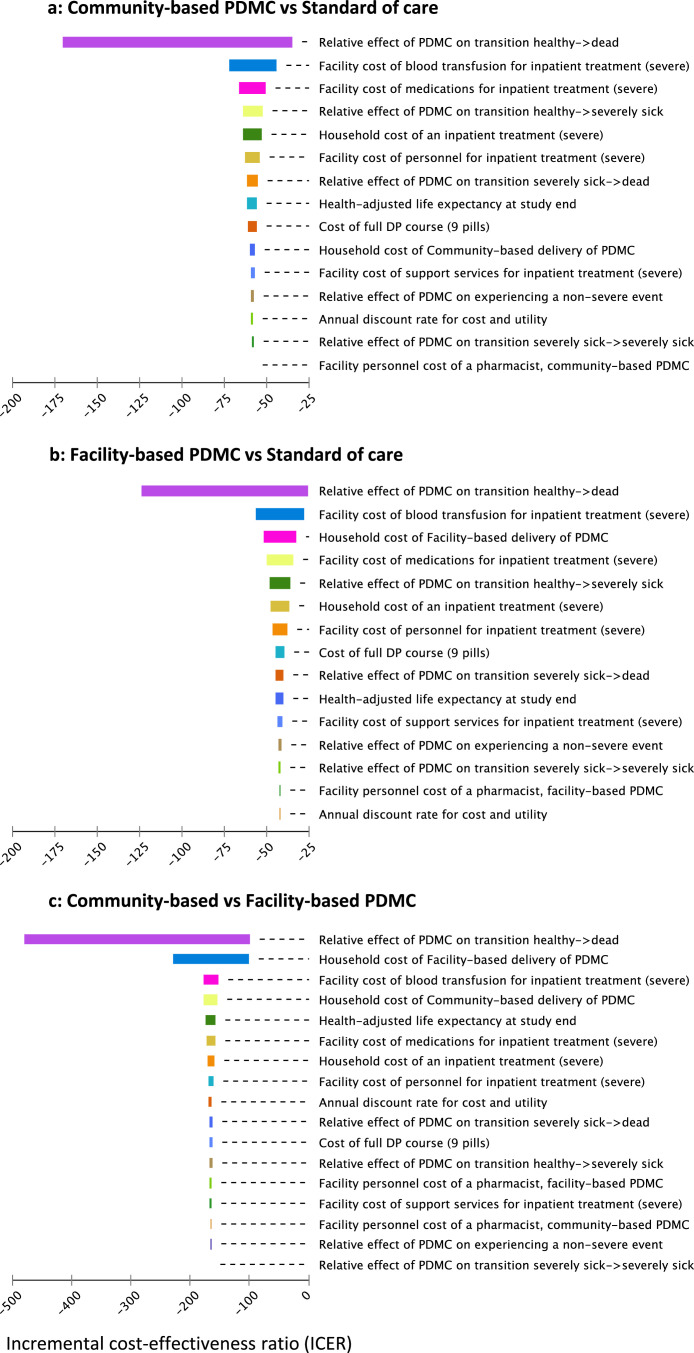
**a-c: Deterministic sensitivity analysis for Malawi; tornado diagram of community-delivered PDMC and facility-delivered PDMC versus standard of care (1a, 1b), and a comparison of both PDMC strategies (1c)**. The three Figure 1a-c combine data from Kwambai (2020), Gondwe (2021), as well as unpublished costing data from Malawi (Tables S4–9).^8^^,^^11^ The baseline strategy is named second in each graph. The variables are sorted according to decreasing sensitivity on the ICER. The ICER is expressed in terms of USD per QALY gained. A willingness to pay-threshold of one gross domestic product (GDP) per capita was included (535 USD in Malawi, 2017). The ICERs shown here are negative as result of the negative cost and positive incremental effects of PDMC. The figures show the potential changes in the overall incremental cost-effectiveness ratio (ICER) that can be achieved when varying single parameters between lower and higher value estimates. No modification in a single variable was influential enough to result in a positive ICER for any of the three two-strategy comparisons. This means that within its parameters, no variable could impact the model to the degree that the respective baseline strategy would become cost-effective. In all comparisons, the probability of dying was the variable with the highest single potential to influence the ICER value. This is explained by the reward used in the model: health-adjusted life expectancy. Any child death results in a complete loss of the life expectancy rewarded to surviving children. This life expectancy, however, decreases only by a relatively small amount when children transition to non-healthy states within the six-months follow-up period. In the comparisons with the standard of care, the cost of blood transfusion is the second most influential parameter. Blood transfusions are less frequent with PDMC-treatment because of the reduction in readmissions compared to standard of care. In addition, a readmitted child with PDMC treatment was less likely to need a blood transfusion than a readmitted child receiving standard of care. Figure 1a and b indicate that community-based PDMC is the better strategy based on the overall ICER, which is partly explained by the higher sensitivity of household costs under facility-based delivery (Figure 1b). PDMC=postdischarge malaria chemoprevention. DP=dihydroartemisinin–piperaquine. ICER= incremental cost-effectiveness ratio. USD=United States Dollars.

Blood transfusion costs were estimated separately due to their significant contribution to the total costs (Figure 1). Laboratory staff estimated that 70% of the blood available at Zomba Central Hospital originated from the central blood bank and 30% from local donations. We used this ratio to estimate blood transfusion costs for Malawi based on the literature on transfusion costs.^22^ For Kenya and Uganda, we relied on WHO cost estimates and the literature.^5^^,^^24^ Approximately 42% of readmissions in the control arm of the efficacy trial required blood transfusions, compared to 29% in the intervention arm (Table S5).^8^ We estimated the average transfusions needed for the different adherence categories using linear interpolation.

Non-severe health events comprised outpatient visits at health centres and hospital outpatient departments. We established the average costs for a non-severe illness by employing the same process as for readmission costs. In the absence of access to patient files, we approximated the average medication costs based on the standard of care for the most frequent diagnosis: uncomplicated clinical malaria (85%).^8^ Support services costs, including information technology, laundry and cleaning, were allocated using the annual share of malaria-related admissions among the paediatrics patients as the allocation key. Maintenance costs were allocated using the surface share of the paediatric inpatient ward and outpatient department as the allocation key (Table S8). Both costs were adopted for Kenya and Uganda. Hospital capital costs were disregarded as all relevant facilities in Malawi were publicly owned and over 30 years old.

Direct household costs and time used for adverse health events were collected from the caregivers of children partaking in the trials. We estimated indirect household costs as productive time lost for the emergency-related time, valued by minimum national salary rates (Table S8). All cost data collected during the trials were converted into USD, using the exchange rates of June 2018. All others were inflation-adjusted to 2018.

### Analysis and uncertainty

Univariate deterministic sensitivity analyses of key input variables were performed using +/- one standard deviation of their mean values. We used +/-50% ranges for point estimates of costs, which typically have larger variation than other data, and +/-25% for other variables where we lacked inference data (Table 1). We also report one-way sensitivity analyses as Tornado diagrams with pairwise comparisons of two strategies.

As explained above, we assumed a linear dose-effect relationship of DP in the base-case analysis, thus a proportionally reduced effect with lower adherence. We conducted scenario analyses for a concave and convex dose-effect curve leading to higher or lower efficacy for the *medium* and *low* adherence categories (Figure S2). We performed probabilistic sensitivity analyses for each country using Monte Carlo simulation with 10 000 iterations. The distribution shapes and confidence intervals determined the analysis parameters where they were available. In their absence, the ranges from the deterministic sensitivity analysis were adopted with standard distributions for costs (gamma) and probabilities (beta).

### Ethics Statement

The data we used was collected as part of two clinical trials with ethical approval, documented elsewhere in detail. The responsible review committees in Kenya, Uganda, the United Kingdom, and Norway approved the efficacy trial.^8^ The implementation trial was approved by review committees in Malawi and Norway.^11^ All approved our use of the trial data for this study.

### Role of the funding source

The funder had no role in study design, collection, analysis, and interpretation of data, or in the writing and submission of this study. MJK and BR had full access to the data and took the decision to submit the results for publication.

## Results

### Cost-effectiveness

From a societal perspective, combining both health care provider and household perspectives, the average expected cost of community-based PDMC per child treated in Malawi, Kenya, and Uganda was 22·74, 37·87, and 29·78 USD, respectively, which represents an average reduction of costs by 49%, 50% and 47% compared to the estimated average cost of the standard of care. Facility-delivered PDMC incurred a smaller reduction of cost by an average of 31%, 35%, and 27%, respectively (Table 2). In both PDMC strategies, the intervention costs of PDMC were more than outweighed by saved costs for readmission.

**Table 2:** Incremental cost-effectiveness ratios per country, comparing community-based postdischarge malaria chemoprevention (PDMC) with facility-based PDMC, and with the national standard of care.

		Cost (USD^a^)				Effectiveness (QALY^b^)		Cost-effectiveness
Country	Strategy	Health care provider cost	Household cost	Total cost	Incremental cost	HALE^c^	Incremental QALY	ICER^d^
Malawi	Standard of care	36·00	8·91	44·84		52·65		negative
	PDMC Facility-delivered	19·50	11·65	31·11	−13·72	52·98	0·33	negative
	PDMC Community-delivered	16·95	5·83	22·74	−8·37	53·03	0·05	dominant
Kenya	Standard of care	46·63	29·98	76·40		53·86		negative
	PDMC Facility-delivered	26·27	23·47	51·49	−24·91	54·20	0·34	negative
	PDMC Community-delivered	22·54	15·72	37·87	−13·61	54·25	0·05	dominant
Uganda	Standard of care	41·95	14·16	56·00		53·84		negative
	PDMC Facility-delivered	22·46	18·44	40·84	−15·16	54·18	0·34	negative
	PDMC Community-delivered	19·33	10·50	29·78	−11·07	54·23	0·05	dominant

Incremental cost-effectiveness rankings per country. This table reports mean values from Monte-Carlo simulations of 10·000 iterations per country. Confidence intervals are shown as 95% confidence interval ellipsoids in Figures 3a-c; an extended version of this table with confidence intervals of the mean values is shown in the supplementary materials, Table S9. When comparing the three strategies, Community-delivered PDMC was the absolute dominant strategy: it was at the same time the least costly over the expected lifetime of a child (lowest cost per QALY gained) and yielded the most health-adjusted life-years. The incremental quality-adjusted life years (QALY) specify each strategy's expected impact on mortality and morbidity. The incremental values indicate that the facility-based distribution also absolutely dominates the standard of care. However, it is less cost-saving and less effective than community-based distribution when compared to standard of care.

a USD– United States Dollar.

b QALY– Quality-adjusted life years.

c HALE– Health-adjusted life expectancy.

d ICER– Incremental cost-effectiveness ratio.

Compared to the standard of care, both community-based and facility-based PDMC resulted in net cost savings for health care providers from the reduced readmissions. These savings were most influenced by the reduced need for blood transfusions and the proportionate reduction in blood transfusions per readmission when using PDMC (Figure S2). Due to its increased adherence, community-based delivery was the least costly delivery strategy for providers. From a household perspective, community-based PDMC compared to the average standard of care costs per child treated resulted in net savings of approximately one-third, one-half, and one-quarter in Malawi, Kenya, and Uganda, respectively. However, facility-based delivery was, on average, more costly to households in Malawi and Uganda than the standard of care, with the monthly drug collection costs outweighing the costs of an increased readmission risk (Table 2).

The differences in effects were relatively less pronounced. PDMC primarily reduces readmissions, and each readmission translated into a reduction of a child's quality of life, lasting one month, in the models. In all three countries, the combination of reduced mortality and morbidity resulted in an expected gain of 0·4 QALY to a child's health-adjusted life expectancy when comparing community-based PDMC to the standard of care. This was 0·3 QALY for facility-based PDMC (Table 2).

Both PDMC strategies were cost-saving as they were less costly and more effective than the standard of care over the lifetime of a child eligible for PDMC. These results were largely driven by cost savings from fewer non-severe and severe adverse events relative to the standard of care. In each country, community-based delivery was the cost-effective strategy. Compared to community-based PDMC, the higher household costs of obtaining PDMC at the hospitals, and the associated lower adherence, made facility-based delivery sub-optimal for PDMC delivery (Table 2).

### Sensitivity Analyses

One-way deterministic sensitivity analyses showed that the effect of PDMC on the probability of dying was the most influential individual determinant on the ICERs for both strategies, explained by the heavy impact of mortality on children's health-adjusted life expectancy, compared to the impact of short-term disability weights for readmissions and non-severe health events (Figure 1). No single parameter was sufficiently influential for facility-based PDMC or the standard of care to become the optimal strategy. Only unrealistically large changes to any single parameter could lead to a conclusion-changing base-case ICER. Univariate sensitivity analysis of the Malawi data showed that community-based delivery was consistently more cost-effective than facility-based delivery. Deterministic sensitivity analysis for Uganda and Kenya showed similar results. Changing the linear dose-effect assumption to convex or concave scenarios did not change the ranking in any of the three countries.

**Figure 2:**
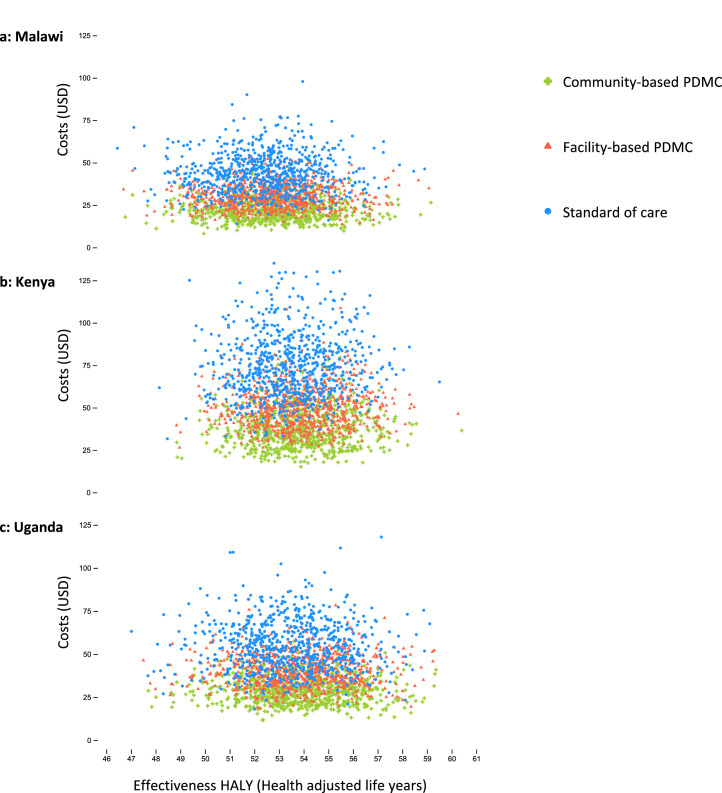
**a-c: Monte Carlo simulation of 750 iterations for cost-effectiveness analysis of the two PDMC strategies and the standard of care in Malawi, Kenya, and Uganda**. We used 10,000 iterations per country model for the general cost-effectiveness and probabilistic sensitivity analyses (Tables 2 and S10). For visualization purposes, we reduced the number of iterations in the above scatterplots. The 750 iterations display 750 independent cost-effectiveness analyses per country, each conducted with probabilistic sampling from the distributions provided (Table 1). The plots thus display 750 times three interrelated cost-effectiveness values, one per strategy. In each country, there is relatively little difference between the three differently coloured intervention “clouds” on the x-axis, “Effectiveness HALY (Health adjusted life years)”. This indicates a relatively small difference in effectiveness between the strategies; however, a weak accumulation of relatively higher effectiveness values can be observed in favour of community-based PDMC delivery (green crosses) over facility-based PDMC delivery (red triangles), over the standard of care (blue dots). The difference in costs between the strategies is more clearly illustrated, shown as the horizontal layering of the clouds along the y-axis (“Costs (USD)”), with community-based PDMC being predominantly less costly than facility-based PDMC than the standard of care. PDMC=postdischarge malaria chemoprevention. DP=dihydroartemisinin–piperaquine. USD=United States Dollars.

**Figure 3:**
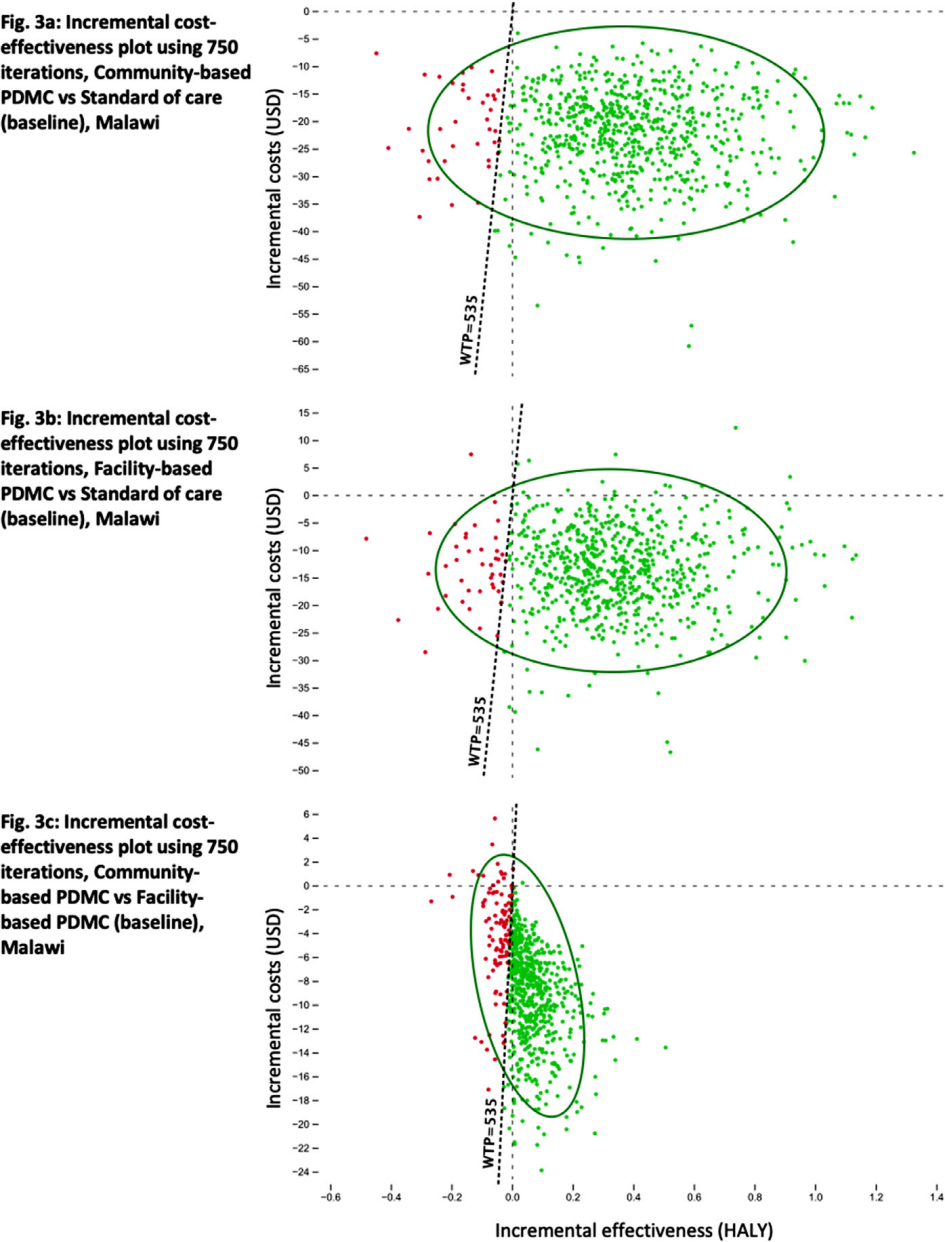
**a-c: Simulation of incremental cost-effectiveness calculations for PDMC in Malawi (750 iterations) with pairwise comparisons of the three strategies, each with a 95% confidence ellipse, and a willingness to pay-line of one GDP per capita in USD (WTP, 535 USD for Malawi, 2017): a) community-based PDMC versus standard of care; b) facility-based PDMC versus standard of care; c) community- versus facility-based PDMC**. In each of the three scatterplots, the expected average cost and effectiveness of the baseline strategy are set as zero USD and zero HALY, respectively, at the intersection of the dotted lines. Each of the 750 dots (red and green) represents the cost and effectiveness of the comparator strategy in 750 iterations. Green dots indicate that the comparator strategy was cost-effective compared to the baseline (East of the WTP) in that particular iteration. Red dots represent iterations where the baseline strategy was found cost-effective (West of the WTP). The green ellipses show the 95% confidence interval. The frequency and proportion of iterations (10·000) per quadrant and category are shown for all countries in the supplementary material (Table S10). PDMC=postdischarge malaria chemoprevention. DP=dihydroartemisinin–piperaquine. USD=United States Dollars.

The probabilistic sensitivity analyses based on Monte Carlo simulations suggested that community-based PDMC is highly likely to be superior to standard care and facility-based PDMC in Malawi (Figure 2). The differences between the strategies’ cost-effectiveness rankings were largely driven by costs, as suggested by the horizontal layering of the strategies’ iteration clusters on the y-axes (Figure 2). Changes in effectiveness were less influential, which is shown in the relatively small differences between clusters on the x-axes (Figure 2). Pairwise comparisons of the strategies’ incremental costs and effectiveness in Malawi were assessed against a willingness-to-pay threshold (WTP) set at one gross domestic product per capita in 2017, i.e. 535 USD (Figure 3). These analyses show that community-based delivery of PDMC with the estimated WTP was cost-effective in 95·3% of our iterations, with 93·6% being superior, i.e. resulting in lower cost and higher effectiveness, compared to the standard of care (Figure 3a, Table S10). In Kenya, at a WTP of 1708 USD, community- and facility-based PDMC were cost-effective compared to standard of care in 94·4% and 94·1% of the iterations. The corresponding figures in Uganda were 94·9% and 94·4% (WTP of 770 USD). Community-based PDMC was the cost-effective PDMC-strategy in 84·9% (Malawi), 82·6% (Kenya) and 85·0% (Uganda) of 10 000 model iterations per country (Table S10).

## Discussion

This cost-effectiveness analysis showed that both PDMC strategies were cost-effective and cost-saving compared to standard of care. They were less costly and more effective in terms of quality-adjusted life-years than the standard of care from a facility and household perspective in all three countries. The main driver of the PDMC dominance is the reduced cost resulting from fewer readmissions in the PDMC arms relative to the standard of care.

Community-delivered PDMC was the most cost-saving of the two strategies because the repeated multiple hour-travels for drug collection in the facility-based strategy presented the caregivers with higher costs and a disincentive to adhere. These results remained robust in the deterministic and probabilistic sensitivity analyses and were consistent across all three countries. The results were also robust to changes in the assumptions about the relationship between adherence and effectiveness. We assumed a linear dose-response because there were no real-life dose-response data about this relationship. We adjusted for this uncertainty through scenario analyses and the probabilistic sensitivity of the models, neither of which changed the cost-effectiveness ranking. Our finding that community-based delivery of PDMC is cost-effective is consistent with healthcare providers’ and caregivers’ preferences as reported in previous qualitative studies from Malawi.^25^^,^^26^

We expect our results to be useful for policy considerations. Establishing the cost-effectiveness of an intervention is essential for informed priority setting and developing benefit packages in a health system. One strength of our analysis is the high internal validity for southeastern Africa by combining the context-specific efficacy estimates from a large placebo-controlled efficacy trial in Uganda and Kenya with strategy-specific adherence data from a delivery mechanism trial in Malawi. By adjusting PDMC's proven efficacy with robust adherence data, we offer a modelling method to tailor cost-effectiveness analyses for greater external validity and policy relevance more broadly.

Limitations include using facility costing data for Kenya and Uganda partly based on data obtained in Malawi. Although we used country-specific unit estimates for personnel costs and the costs of blood transfusions to control for the largest share of between-country differences, some directly adopted costs may result in inaccurate estimates. Furthermore, we used standardised ranges for sensitivity analysis of the cost components for which inference data were lacking. Lastly, our analysis does not consider the health systems’ costs at the regional and national levels of introducing PDMC. PDMC, unlike intermittent preventive treatment in infants or pregnancy, does not have an existing platform through which it can be delivered, and new delivery strategies and country-specific implementation modes must be considered. Future research comparing the country-specific implementation cost and exploring the underlying structural factors may provide additional support to national health systems’ implementation efforts.

PDMC is a relatively simple intervention with a high potential of being cost-saving because it is less costly and more effective in increasing health-adjusted life expectancy than the current standard of care in Kenya, Uganda, and Malawi. In addition, providing all PDMC courses to the caregiver at discharge, combined with instructions on administering them, is less costly for providers and households and more effective than a facility-based delivery that requires the caregiver to collect each monthly dose of PDMC.

## Contributors

MJK was responsible for the conceptualisation and methodology, data curation and verification, software use, formal analysis, the writing of the original draft, visualisation of results, and the editing of the manuscript. BR was responsible for the conceptualisation and methodology, he verified and validated the data, contributed to software use and formal analysis, as well as the writing of the original draft, results visualisation, and manuscript editing. KSP has verified the underlying data, was responsible for validation and investigation, and contributed to the review and editing of the manuscript. BR and KSP together supervised and administrated the project behind this study and aquired the funding for it. FTK was responsible for the conceptualisation, the data verification, investigation, the review and editing of the manuscript, and aquired the project funding. RO is responsible for data curation, validation, investigation, review and editing of the manuscript, as well as funding acquisition. TNG, AD, TKK, ATM, CCJ, RI contributed in the data curation, the validation and investigation, and in the editing and review of this manuscript. In addition, TNG, AD, and TKK also verified the underlying data. MJK and BR had full access to the dataset and accept the responsibility to submit this study for publication.

## Data sharing statement

This study did not use individual participant data. We used summarised data from two trials that shared data according to the requirements of the International Committee of Medical Journal Editors.^8^^,^^11^^,^^27^^,^^28^

## Declaration of interests

There are no conflicts of interest to declare.

## Appendix Supplementary materials

Image, application 1
