# Supplementary material for: Economic evaluation of postdischarge malaria chemoprevention in preschool children treated for severe anaemia in Malawi, Kenya, and Uganda: A cost-effectiveness analysis
Source: eClinicalMedicine. 2022 Oct 1;52:101669. doi: 10.1016/j.eclinm.2022.101669 (PMC9596312; doi:10.1016/j.eclinm.2022.101669)
Supplement: Supplementary file 1 [file mmc1.pdf]

Annex: Supplementary materials

Kühl et al.

Economic evaluation of postdischarge malaria chemoprevention in preschool children treated for severe anaemia: cost-effectiveness analyses for Malawi, Kenya, and Uganda.

Table of Contents

Supplementary Figures

Figure S1: Overview of our decision model, exemplary for Malawi 3

Figure S2: Linear extrapolation of dose-effect (DE) estimates of PDMC by adherence category 3

Supplementary Tables

Table S1: The five arms used in the implementation trial, pooled in two arms 4

Table S2: Transition probabilities between the health states *healthy*, *severely sick,* and *dead* 4

Table S3: Medication and medical equipment costs for intervention, readmission, non-severe treatment 5

Table S4: Facility personnel costs for intervention, readmission, non-severe treatment 6

Table S5: Medication costs overview used for severe anaemia treatment in preschool children, Malawi 7

Table S6: Support service costs based on annual expenses of Zomba Central Hospital, Malawi 8

Table S7: Household costs for PDMC intervention 9

Table S8: Household costs for hospital readmission, non-severe treatment 10

Table S9 Incremental cost-effectiveness ratios per country 11

Table S10 Pairwise cost-effectiveness comparison of the two PMDC strategies and the standard of care 12

References

Supplementary Figures

Figure S1: Overview of our decision model, exemplary for Malawi, with a decision tree to control adherence and a Markov model to control health outcomes over the follow-up period of six months.

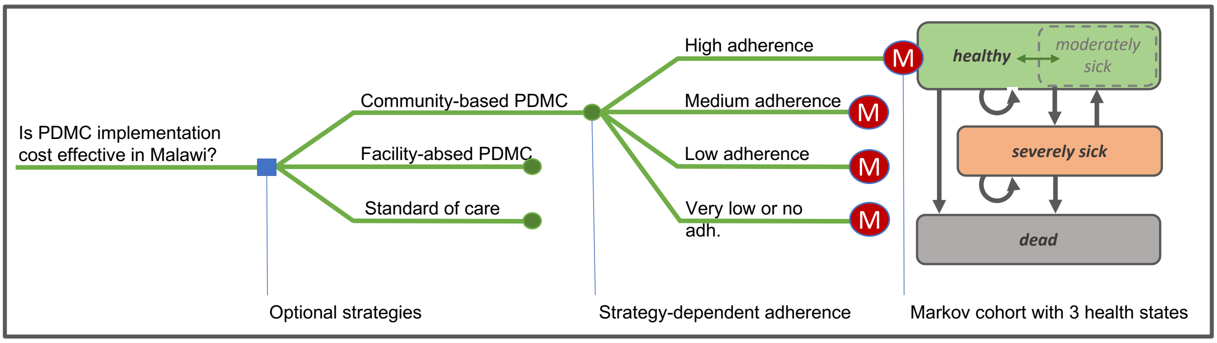

Figure S2: Linear extrapolation of dose-effect (DE) estimates of PDMC by adherence category, including scenario analyses for a convex and a concave dose-effect curve between zero (standard of care) and full efficacy of PDMC.

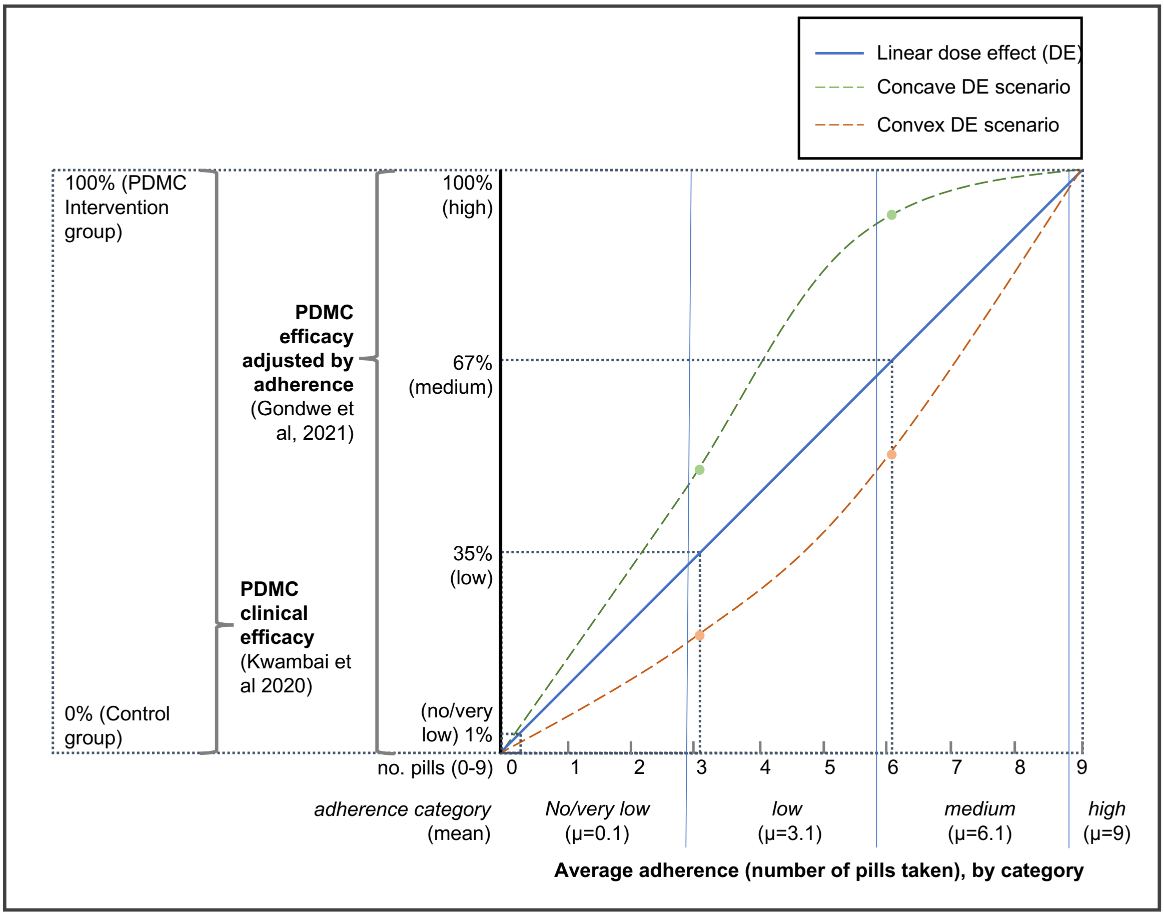

On the left of the graph, the efficacy range for PDMC-adherence is defined by the protective effect of PDMC on child mortality and readmission rate (100%) compared to the placebo/standard of care efficacy (0%).[1] Based on the mean values for pills given per adherence category, we extrapolated the efficacy of the categories *no or very low*, *low*, and *medium* adherence.[2] High adherence is equated to the full intervention effect of the efficacy trial (100%). Further, we assumed a concavely-shaped (+20%, green) and convexly-shaped (-20%, orange) dose-effect within the same range, thus not changing the efficacy of full adherence.

Abbreviations: PDMC=postdischarge malaria chemoprevention.

Supplementary Tables

Table S1: The five arms used in the implementation trial (Gondwe et al., 2021) were pooled in two arms: community- and facility-based PDMC delivery.[2] The factorial allocation in the trial added reminder options combining SMS-reminder with in-person-reminders by village-based Health Surveillance Assistants (HSA).

Category of adherence	Community-based delivery of PDMC					Facility-based delivery of PDMC			
	3 arms in Gondwe (2021)					2 arms in Gondwe (2021)			
	Com-SMS	Com+SMS	Com+HSA	pooled total	(%)	Fac-SMS	Fac+SMS	pooled total	percent
No or very low	1	2	2	5	2·3	4	2	6	4·0
Low	8	4	6	18	8·1	12	14	26	17·3
Medium	16	9	17	42	19·0	21	19	40	26·7
High	43	59	54	156	70·6	39	39	78	52·0
Total	68 (100)	74 (100)	79 (100)	221	100·0	76 (100)	74 (100)	150·0	100·0

Abbreviations: PDMC=postdischarge malaria chemoprevention. Com=community-based delivery of PDMC. Fac=facility-based delivery of PDMC. +SMS/-SMS=with/without SMS reminder.

Table S2: Transition probabilities between the health states *healthy*, *severely sick,* and *dead* for the standard of care arm and the relative risk of transitions with PDMC treatment, derived from the PDMC efficacy trial (Kwambai et al., 2020).[1]

Starting state	Trial arm allocation	Transition state		
		healthy	*severely sick*	*dead*
healthy	Standard of Care (base case)	0·8710	0·1238	0·0051
	Relative risk with PDMC	1·0954	0·3391	0·7490
severely sick	Standard of Care (base case)	0·9043	0·0891	0·0066
	Relative risk with PDMC	1·0121	0·8738	1·0472
dead	Standard of Care (base case)	-	-	1
	Probability PDMC	-	-	1

Abbreviations: PDMC=postdischarge malaria chemoprevention.

Table S3: Medication and medical equipment costs for intervention, readmission, and non-severe treatment (Clinic/OPD) in Malawi, Kenya, and Uganda. This data was collected alongside the implementation trial (Gondwe, 2021) and has not been separately published.[2]

			Malawi (USD)				Kenya (USD)				Uganda (USD)			
	**Medication and medical equipment components**	**average units needed for standard treatment, based on Malawi data**	**cost per unit**	**mean cost per child**	**lower limit**	**upper limit**	**cost per unit**	**mean cost per child**	**lower limit**	**upper limit**	**cost per unit**	**mean cost per child**	**lower limit**	**upper limit**
**PDMC Intervention**	dihydroartemisinin-piperaquine	9	0·33	2·97	1·48	4·45	0·25	2·25	1·13	3·38	0·26	2·30	1·15	3·45
**Readmission**	average sum medication for SA treatment: see Table S6	n/a; see Table S6	n/a	15·03	7·52	22·55	see Malawi				see Malawi			
	blood transfusion, placebo-arm, efficacy trial (Kwambai 2020))	0·42	65·93	27·69	13·85	41·54	73·10	30·70	15·35	46·05	82·40	34·61	17·72	51·91
	blood transfusion, PMC-arm, efficacy trial (Kwambai 2020))	0·29	65·93	19·38	9·69	29·08	73·10	21·49	10·75	32·24	82·40	24·23	12·41	36·34
**Clinic/OPD**	standard of care medication, clinical malaria: artemether/lumefanterine	6	0·14	0·85	0·43	1·28	see Malawi				see Malawi			

Abbreviations: PDMC=postdischarge malaria chemoprevention. USD=United States Dollars. Clinic/OPD=Summary category for visits to local clinics and health centres as well as the outpatient department of the hospital (OPD).

Table S4: Facility personnel costs for intervention, readmission, and non-severe treatment (Clinic/OPD), based on hospital practice in Malawi. This data was collected alongside the implementation trial (Gondwe, 2021) and has not been separately published.[2]

			Malawi (USD)		Kenya (USD)		Uganda (USD)	
	**Hospital employee position**	**Average time spent per child based on observations in Zomba Central Hospital, Malawi (hours)**	**Hourly personnel cost**	**Personnel cost per child treated with PDMC**	**Hourly personnel cost**	**Personnel cost per child treated with PDMC**	**Hourly personnel cost**	**Personnel cost per child treated with PDMC***
**PDMC Intervention**	Pharmacist, community-based PDMC	0·08	1·43	0·12	5·75	0·48	4·61	0·38
	Pharmacist, facility-based PDMC	0·17	1·43	0·24	5·75	0·96	4·61	0·77
**Readmisson**	Clinician	0·87	3·25	2·81	7·10	6·15	6·60	5·72
	Nurse	1·83	2·85	5·23	3·46	6·34	1·46	2·67
	Pharmacist	0·17	1·43	0·24	5·75	0·96	4·61	0·77
	patient attendant	0·42	0·97	0·41	2·43	1·01	0·02	0·01
	clerk	0·18	1·03	0·19	2·57	0·47	1·54	0·28
	**average total personnel cost per child treated**			**8·87**		**14·94**		**9·46**
**Clinic/OPD**	Clinician	0·08	3·25	0·27	7·10	0·59	6·60	0·55
	Nurse	0·33	2·85	0·95	3·46	1·15	1·46	0·49
	Pharmacist	0·08	1·43	0·12	5·75	0·48	4·61	0·38
	patient attendant	0·08	0·97	0·08	2·43	0·20	0·02	0·00
	clerk	0·17	1·03	0·17	2·57	0·43	1·54	0·26
	**average total personnel cost per child treated**			**1·59**		**2·85**		**1·68**

Abbreviations: PDMC=postdischarge malaria chemoprevention. USD=United States Dollars. Clinic/OPD=Summary category for visits to local clinics and health centres and the outpatient department of the hospital (OPD).

Table S5: Medication overview used for severe anaemia (SA) treatment in Malawi in preschool children, derived from a sample of 50 children. Cost shown in Malawi Kwacha (MKW) and total in US Dollars (USD). This data was collected alongside the implementation trial (Gondwe, 2021) and has not been separately published.[2]

Medication for inpatient treatment	Unit price (MKW)	Average units needed per child if indicated	Cost if indicated (MKW)	Costs with additional wastage and freight (30%)	Proportion of children with indication	Average total per SA treatment (MKW)	Average total per SA treatment (USD)
**Medication SA treatment**							
Albendazole, 200 milligrams (mg)	6·42	2·00	12·84	16·98	0·30	5·09	0·01
Amoxycillin, 125mg/5milliliters (ml) suspension	676·40	1·00	676·40	894·54	0·05	44·73	0·06
Artesunate, 60mg with diluent	1472·00	3·00	4416·00	5840·16	1·03	5986·16	8·20
Benzylpenicillin, 3 grams (g)	149·00	13·00	1937·00	2561·68	0·48	1216·80	1·67
Ceftriaxone, 1g	178·00	5·00	890·00	1177·03	0·13	153·01	0·21
Dextrose 50%, 50ml	457·00	1·00	457·00	604·38	0·08	48·35	0·07
Diazepam 5mg/ml, 2ml	121·00	1·00	121·00	160·02	0·03	4·00	0·01
Furosemide (Frusemide) 10mg/ml, 2ml	40·00	1·00	40·00	52·90	0·05	2·65	0·00
Gentamycin Sulphate 40mg/ml, 2ml	43·00	5·00	215·00	284·34	0·33	92·41	0·13
Ferrous sulphate 200mg/folic acid 250 micrograms, coated tablets	2·25	30·00	67·56	89·35	0·08	6·70	0·01
Artemether-lumefantrine, 20mg-120mg	37·76	6·00	226·56	299·63	0·53	157·30	0·22
Nystatin oral suspension 100,000 IU/ml, 20ml	399·00	1·00	399·00	527·68	0·13	65·96	0·09
Paracetamol syrup 120mg/5ml, 100ml	313·00	9·00	2817·00	3725·48	0·85	3166·66	4·34
Phenobarbitone sodium 200mg/ml, 1ml	637·00	1·00	637·00	842·43	0·03	21·06	0·03
Zinc sulphate 20mg, tablets	4·50	10·00	45·00	59·51	0·03	1·49	0·00
**Average total medication cost for SA treatment per child**						**10972·37**	**15·03**
**Medication at discharge**							
Albendazole, 200mg	6·42	2·00	12·84	16·98	0·70	11·88	0·02
Artemether-lumefantrine, 20mg-120mg	37·76	6·00	226·56	299·63	0·48	142·32	0·19
Ferrous sulphate 200mg/folic acid 250 micrograms, coated tablets	2·25	30·00	67·56	89·35	0·93	82·65	0·11
**Average discharge cost for SA treatment per child**						**236·85**	**0·32**

Table S6: Support service costs based on annual expenses of Zomba Central Hospital, Malawi, separated in inpatient paediatric department cost (severe health event) and outpatient department or clinic visits for non-severe events. This data was collected alongside the implementation trial (Gondwe 2021) and has not been separately published.[2] It was adopted for Kenya and Uganda, and adjusted for average hospital days of children readmitted in the efficacy trial (Kwambai, 2020).[1]

Facility running cost and support services	Total expenses 2017-18 (MKW)	Proportion of the inpatient paediatric ward (IPD) of the entire hospital*	Proportion of outpatient paediatric department (OPD) of the entire hospital*	Estimated annual cost, IPD (MKW)	Estimated annual cost, OPD (MKW)	IPD daily patient cost (6076 patients/year)	Patient cost for full SA treatment (Malawi: 4·66 days)	OPD per patient-cost (9114 patients/year, MKW)
Medical Equipment	5000000·00	0·18	0·04	900000·00	180000·00	148·12	690·26	44·44
Building rehabilitation	5500000·00	0·18	0·04	990000·00	198000·00	162·94	759·28	48·88
Maintenance	12000000·00	0·18	0·04	2160000·00	432000·00	355·50	1656·62	106·65
Internet	7200000·00	0·18	0·04	1296000·00	259200·00	213·30	993·97	63·99
Water	43000000·00	0·18	0·04	7740000·00	1548000·00	1273·86	5936·21	382·16
Landscaping	12000000·00	0·18	0·04	2160000·00	432000·00	355·50	1656·62	106·65
Security service	15000000·00	0·18	0·04	2700000·00	540000·00	444·37	2070·77	133·31
Total cost per patient (MKW)						2953·59	13763·72	590·72
Total cost per patient (USD)						4·05	18·85	0·81
Total cost per patient, Kenya (average inpatient duration: 5·54 days, USD)							22·41	0·96
Total cost per patient, Uganda (average inpatient duration: 3·86 days, USD)							15·62	0·67

*The proportion was determined as the departments' respective share of the hospitals overall surface area (buildings only).

Abbreviations: SA=Severe anaemia. PDMC=postdischarge malaria chemoprevention. MKW=Malawi Kwacha. USD=United States Dollars.

Table S7: Household costs for PDMC intervention. The data on households' economic costs from Malawi was collected alongside the implementation trial (Gondwe 2021) and has not been separately published.[2] Data for Kenya and Uganda was collected alongside the efficacy trial and has neither been published (Kwambai, 2020).[1]

**Household Cost: PDMC Intervention**									
	**Uganda**			**Kenya**			**Malawi**		
	mean	*CI*		*mean*	*CI*		*mean*	*CI*	
		lower	*upper*		*lower*	*upper*		*lower*	*upper*
Financial cost due to administering PDMC at home (UGX, KES, MKW)	10820·48	8516·66	13124·29	6·46	1·24	11·68	94·26	41·21	147·31
Time spent administering full treatment, 9 tablets (Minutes)	138·70	127·69	127·69	178·57	159·42	197·72	258·00	85·36	430·64
Equivalent minimum salary, 8·5 hrs/day (UGX, KES, MKW)	1414·21	1301·89	1301·89	190·09	169·70	210·47	680·92	225·29	1136·54
Total cost intervention (UGX, KES, MKW)	12234·69	9818·56	14426·19	196·55	170·94	222·15	775·18	266·50	1283·86
**Total cost intervention (USD)**	**3·31**	**2·65**	**3·90**	**1·94**	**1·68**	**2·19**	**1·07**	**0·37**	**1·78**
**Household Cost of DHP collection at hospital (facility-based delivery)**									
Money spent on transport (UGX, KES, MKW)	16146·13	15564·72	16727·53	362·30	332·31	392·29	1818·06	1557·95	2078·18
Duration of transportation and hospital visit (hours)	4·20	4·04	4·36	2·22	2·11	2·32	5·34	4·94	5·75
Equivalent minimum salary, 8·5 hrs/day (UGX, KES, MKW)	21841·49	21027·12	22655·86	1203·72	1147·04	1260·40	7193·85	6650·37	7737·34
Total costs for collection (UGX, KES, MKW)	37987·62	36591·84	39383·39	1566·02	1479·35	1652·69	9011·92	8208·32	9815·51
**Total costs for collection (USD)**	**10·27**	**9·89**	**10·64**	**15·43**	**14·57**	**16·28**	**12·46**	**11·35**	**13·58**

Abbreviations: PDMC=postdischarge malaria chemoprevention. UGX=Ugandan Shilling. KES=Kenyan Shilling. MKW=Malawi Kwacha. USD=United States Dollars. CI=Confidence interval.

Table S8: Household costs for hospital readmission and treatment of non-severe cases. The data on households' economic costs from Malawi was collected alongside the implementation trial (Gondwe 2021) and has not been separately published.[2] Data for Kenya and Uganda was collected alongside the efficacy trial and has neither been published (Kwambai, 2020).[1]

**Household costs: Hospital readmission**									
	**Uganda**			**Kenya**			**Malawi**		
	mean	*CI*		*mean*	*CI*		*mean*	*CI*	
		lower	*upper*		*lower*	*upper*		*lower*	*upper*
Time spent in the hospital (days)	5·80	5·31	6·29	6·32	4·86	7·78	5·05	4·64	5·46
Equivalent minimum salary, 8·5 hrs/day (UGX, KES, MKW)	30162·35	27636·81	32683·80	3430·97	2638·34	4223·59	6803·34	6251·38	7355·29
Money spent at the hospital (UGX, KES, MKW)	24381·88	21204·01	27559·74	446·54	223·27	669·81	99·32	37·94	160·71
Money spent on transport (UGX, KES, MKW)	15947·08	14752·78	17141·37	602·50	384·37	820·63	1818·06	1557·95	2078·18
Total costs of readmission (UGX, KES, MKW)	70491·31	63593·60	77384·91	4480·01	3245·98	5714·03	8720·73	7847·28	9594·18
**Total costs of readmission (USD)**	**19·05**	**17·19**	**20·91**	**44·14**	**31·98**	**56·30**	**12·06**	**10·85**	**13·27**
**Household costs: Outpatient department or clinic visit for a non-severe event**									
	**Uganda**			**Kenya**			**Malawi**		
	mean	*CI*		*mean*	*CI*		*mean*	*CI*	
		lower	*upper*		*lower*	*upper*		*lower*	*upper*
Time spent by caregiver and partner for caring at home, transport to the clinic, time at the clinic (hours)	29·42	27·17	31·68	26·63	24·42	28·84	26·48	22·51	30·46
Equivalent min· salary, 8·5 hrs/day (UGX, KES, MKW)	8516·94	7136·45	9896·06	710·89	569·53	851·88	1739·10	1346·00	2368·25
Money spent at the hospital (UGX, KES, MKW)	7149·06	4982·98	9315·13	98·21	49·11	147·32	21·31	8·14	34·49
Money spent on transport (UGX, KES, MKW)	19859·74	15564·72	16727·53	362·30	332·31	392·29	1818·06	1557·95	2078·18
Total costs of clinic visit (UGX, KES, MKW)	35525·73	27684·15	35938·73	1171·40	950·94	1391·48	3578·47	2912·09	4480·91
**Total costs of clinic visit (USD)**	**9·60**	**7·48**	**9·71**	**11·54**	**9·37**	**13·71**	**4·95**	**4·03**	**6·20**

Abbreviations: PDMC=postdischarge malaria chemoprevention. UGX=Ugandan Shilling. KES=Kenyan Shilling. MKW=Malawi Kwacha. USD=United States Dollars. CI=Confidence interval.

Table S9 Incremental cost-effectiveness ratios per country, comparing community-based postdischarge malaria chemoprevention (PDMC) with facility-based PDMC, and with the national standard of care.

Country	Strategy	**Cost (USD)**				**Effectiveness (QALY)**		**Cost-effectiveness**
		Health care provider cost (mean, 95% CI)	Household cost (mean, 95% CI)	Total cost (mean, 95% CI)	Incremental cost (mean values)	HALE (mean, 95% CI)	Incremental QALY (mean values)	ICER
**Malawi**	Standard of care	36·00 (35·78 - 36·21)	8·91 (8·83 - 8·99)	44·84 (44·61 - 45·07)		52·65 (52·61 - 52·69)		negative
	PDMC Facility-delivered	19·50 (19·38 - 19·62)	11·65 (11·57 - 11·73)	31·11 (30·97 - 31·26)	-13·72	52·98 (52·94 - 53·02)	0·33	negative
	PDMC Community-delivered	16·95 (16·84 - 17·06)	5·83 (5·78 - 5·87)	22·74 (22·62 - 22·87)	-8·37	53·03 (53·00 - 53·07)	0·05	dominant
**Kenya**	Standard of care	46·63 (46·38 - 46·87)	29·98 (29·71 - 30·26)	76·40 (76·02 - 76·77)		53·86 (53·83 - 53·89)		negative
	PDMC Facility-delivered	26·27 (26·13 - 26·41)	23·47 (23·30 - 23·63)	51·49 (51·25 - 51·73)	-24·91	54·20 (54·17 - 54·23)	0·34	negative
	PDMC Community-delivered	22·54 (22·42 - 22·67)	15·72 (15·57 - 15·85)	37·87 (37·66 - 38·08)	-13·61	54·25 (54·22 - 54·28)	0·05	dominant
**Uganda**	Standard of care	41·95 (41·70 - 42·20)	14·16 (14·03 - 14·28)	56·00 (55·71 - 56·29)		53·84 (53·81 - 53·88)		negative
	PDMC Facility-delivered	22·46 (22·33 - 22·60)	18·44 (18·32 - 18·56)	40·84 (40·66 - 41·03)	-15·16	54·18 (54·14 - 54·22)	0·34	negative
	PDMC Community-delivered	19·33 (19·21 - 19·46)	10·50 (10·43 - 10·57)	29·78 (29·62 - 29·93)	-11·07	54·23 (54·20 - 54·27)	0·05	dominant

Abbreviations: PDMC=postdischarge malaria chemoprevention. USD=United States Dollar. QALY=Quality-adjusted life years. 95% CI=95% confidence interval with upper and lower limits in brackets. HALE=Health-adjusted life expectancy. ICER=Incremental cost-effectiveness ratio.

Table S10 Probabilistic sensitivity analysis: Incremental cost-effectiveness report of in pairwise comparison of the two PMDC strategies and standard of care for each country-model, using 10.000 iterations. (see Figure 3a-c for a scatterplot Figure using the Malawi model and 750 iterations).

				Malawi			Kenya			Uganda		
**Component**	**Quadrant**	**Incremental effectiveness (IE)**	**Incremental cost (IC)**	**Incremental cost-effectiveness**	**Frequency**	**Proportion**	**Incremental cost-effectiveness**	**Frequency**	**Proportion**	**Incremental cost-effectiveness**	**Frequency**	**Proportion**
**Community- based PDMC vs. Standard of Care (baseline)**												
C1	IV	IE>0	IC<0	Superior	9365	0.9365	Superior	9337	0.9337	Superior	9353	0.9353
C2	I	IE>0	IC>0	ICER<535·0	0	0	ICER<1708·0	0	0	ICER<770·0	0	0
C3	III	IE<0	IC<0	ICER>535·0	166	0·0166	ICER>1708·0	103	0·0103	ICER>770·0	139	0·0139
C4	I	IE>0	IC>0	ICER>535·0	0	0	ICER>1708·0	0	0	ICER>770·0	0	0
C5	III	IE<0	IC<0	ICER<535·0	469	0·0469	ICER<1708·0	560	0·056	ICER<770·0	508	0·0508
C6	II	IE<0	IC>0	Inferior	0	0	Inferior	0	0	Inferior	0	0
**Facility-based PDMC vs· Standard of Care (baseline)**												
C1	IV	IE>0	IC<0	Superior	9169	0·9169	Superior	9244	0·9244	Superior	8868	0·8868
C2	I	IE>0	IC>0	ICER<535·0	192	0·0192	ICER<1708·0	89	0·0089	ICER<770·0	478	0·0478
C3	III	IE<0	IC<0	ICER>535·0	135	0·0135	ICER>1708·0	75	0·0075	ICER>770·0	93	0·0093
C4	I	IE>0	IC>0	ICER>535·0	0	0	ICER>1708·0	0	0	ICER>770·0	1	0·0001
C5	III	IE<0	IC<0	ICER<535·0	496	0·0496	ICER<1708·0	586	0·0586	ICER<770·0	520	0·052
C6	II	IE<0	IC>0	Inferior	8	0·0008	Inferior	6	0·0006	Inferior	40	0·004
**Community-based PDMC vs· Facility-based PDMC (baseline)**												
C1	IV	IE>0	IC<0	Superior	7737	0·7737	Superior	7766	0·7766	Superior	7754	0·7754
C2	I	IE>0	IC>0	ICER<535·0	13	0·0013	ICER<1708·0	22	0·0022	ICER<770·0	41	0·0041
C3	III	IE<0	IC<0	ICER>535·0	737	0·0737	ICER>1708·0	476	0·0476	ICER>770·0	704	0·0704
C4	I	IE>0	IC>0	ICER>535·0	1	0·0001	ICER>1708·0	2	0·0002	ICER>770·0	4	0·0004
C5	III	IE<0	IC<0	ICER<535·0	1303	0·1303	ICER<1708·0	1516	0·1516	ICER<770·0	1280	0·128
C6	II	IE<0	IC>0	Inferior	209	0.0209	Inferior	218	0.0218	Inferior	217	0.0217

The quadrants are populated according to comparator strategies' cost and effectiveness relative to the average values of the respective baseline-strategy. The categories are defined by these quadrants and additionally limited, where there is no absolute superiority or inferiority, by the willingness to pay threshold. The number of iterations per quadrant and category is listed under "frequency".

Abbreviations: PDMC=postdischarge malaria chemoprevention. ICER=Incremental cost-effectiveness ratio.

References

1. Kwambai T, Dhabangi A, Idro R, et al. Malaria Chemoprevention in the Postdischarge Management of Severe Anemia. *N Engl J Med* 2020; **383**: 2242–54.
2. Gondwe T, Robberstad B, Mukaka M, et al. Adherence to community versus facility-based delivery of monthly malaria chemoprevention with dihydroartemisinin-piperaquine for the post-discharge management of severe anemia in Malawian children: A cluster randomized trial. *PLOS ONE* 2021; **16**(9): e025576.
